# Introducing a Fresh Cadaver Model for Ultrasound-guided Central Venous Access Training in Undergraduate Medical Education

**DOI:** 10.5811/westjem.2016.3.30069

**Published:** 2016-05-05

**Authors:** Ryan Miller, Hang Ho, Vivienne Ng, Melissa Tran, Douglas Rappaport, William J.A. Rappaport, Stewart J. Dandorf, James Dunleavy, Rebecca Viscusi, Richard Amini

**Affiliations:** * College of Medicine, The University of Arizona, Tucson, Arizona; † Department of Emergency Medicine, The University of Arizona, Tucson, Arizona; ‡ Department of Emergency Medicine, Beth Israel Deaconess, Boston, Massachusetts; § Department of Surgery, The University of Arizona, Tucson, Arizona

## Abstract

**Introduction:**

Over the past decade, medical students have witnessed a decline in the opportunities to perform technical skills during their clinical years. Ultrasound-guided central venous access (USG-CVA) is a critical procedure commonly performed by emergency medicine, anesthesia, and general surgery residents, often during their first month of residency. However, the acquisition of skills required to safely perform this procedure is often deficient upon graduation from medical school. To ameliorate this lack of technical proficiency, ultrasound simulation models have been introduced into undergraduate medical education to train venous access skills. Criticisms of simulation models are the innate lack of realistic tactile qualities, as well as the lack of anatomical variances when compared to living patients. The purpose of our investigation was to design and evaluate a life-like and reproducible training model for USG-CVA using a fresh cadaver.

**Methods:**

This was a cross-sectional study at an urban academic medical center. An 18-point procedural knowledge tool and an 18-point procedural skill evaluation tool were administered during a cadaver lab at the beginning and end of the surgical clerkship. During the fresh cadaver lab, procedure naïve third-year medical students were trained on how to perform ultrasound-guided central venous access of the femoral and internal jugular vessels. Preparation of the fresh cadaver model involved placement of a thin-walled latex tubing in the anatomic location of the femoral and internal jugular vein respectively.

**Results:**

Fifty-six third-year medical students participated in this study during their surgical clerkship. The fresh cadaver model provided high quality and lifelike ultrasound images despite numerous cannulation attempts. Technical skill scores improved from an average score of 3 to 12 (p<0.001) and procedural knowledge scores improved from an average score of 4 to 8 (p<0.001).

**Conclusion:**

The use of this novel cadaver model prevented extravasation of fluid, maintained ultrasound-imaging quality, and proved to be an effective educational model allowing third-year medical students to improve and maintain their technical skills.

## INTRODUCTION

Over the past decade, medical students have witnessed a decline in the opportunities to perform technical skills during their clinical years.^1^,^2^ Although the reasons for this diminution in procedural training are complex, it has led to medical students matriculating into residency with a shortage of exposure to common technical skills.^3^,^4^ As a result, simulation-based medical education has come to the forefront to provide training opportunities in a safe environment prior to patient encounters.^5^,^6^ Despite the level of quality that simulation can provide, the tactile and anatomic differences between manikin, phantom models, and the authentic human body make the latter a more attractive training modality.^7^,^8^

In recent years, literature reports on cadaver models for training in central venous cannulation have emerged.^9^–^11^ Though cadaver models improve intravenous cannulation simulation, free flow of fluid into a cannulated post-mortem vessel leads to extravasation of fluid thereby eliminating the cadaver’s utility for other procedures.^10^ In addition, extravasation of fluid results in a distortion of normal anatomy and eventual deterioration of ultrasound image quality.^11^ The purpose of our investigation was to design and evaluate a life-like and reproducible training model for ultrasound-guided central venous access (USG-CVA) using a fresh cadaver.

## METHODS

This was a single-center cross-sectional study conducted at an academic medical center. The study was reviewed by the institutional review board and determined to be exempt. The study participants were 56 third-year medical students without training in USG CV placement. The population had an average age of 29 of which 47% were men and 53% were female. The hands-on fresh cadaver procedure-lab was conducted at the beginning of the surgical clerkship and again five weeks later, and at our institution, the cadaver procedure lab is the first formal training session for USG CV placement. Participation in this study was voluntary and data was gathered from January 2015 to August 2015. All bodies used in our fresh cadaver lab were provided by the College of Medicine Willed Body Program and selflessly donated by our donors and their families.

### Model Design

Equipment necessary includes the following: a marking pen, number 10 scalpel, latex tubing, free ties of 0 silk suture, Toomey syringe, water, red dye, 18-gauge and 22-gauge needles, guide wires, and US with a linear probe. Latex tubing that measures 5/16″ outer diameter, 1/4″ inner diameter with 1/32″ thickness is used to construct the femoral vein model; a smaller latex tubing that measures 7/32″ outer diameter, 5/32″ inner diameter with 1/32″ thickness is used for the internal jugular vein.

#### Femoral Vein (FV) Model

**Figure 1:**
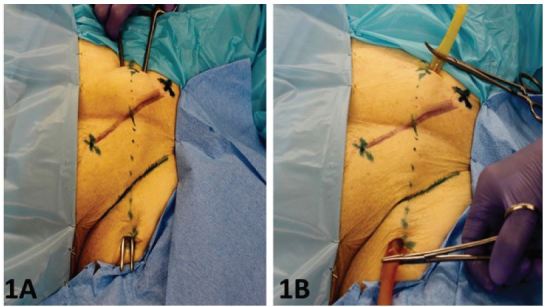
1A) Femoral line model: The clamp is tunneled into the deep subcutaneous tissue midway between the ASIS and pubic tubercle, the bony landmarks are annotated by X’s; 1B) The latex tube has been tunneled into the femoral tissues immediately overlying the femoral sheath to replicate the native vessel anatomy. *ASIS*, anterior superior iliac spine

First, cut a 12-inch length of latex tubing. Use the 0 suture to tie off one end of the tubing so that injected fluid is confined to the tubing. An “X” is placed halfway between the anterior superior iliac spine and the pubic tubercle. This represents the approximate location of the femoral artery (FA) with the femoral vein (FV) lying approximately one centimeter medial. (Six to seven cm above the “X” a three-cm transverse incision, is made down to the external oblique fascia. A similar transverse incision is made about 5cm below the inguinal ligament and likewise carried down to fascia. A long clamp is used to tunnel from the superior to inferior incision (Figure 1A). Once the tip of the clamp is seen, the open end of the latex tubing is placed in between the jaws of the clamp and pulled out from the superior wound. The tubing lies immediately on top of the femoral vein. As seen in figure 1B, by using a longer length of tubing than necessary one can pull the tube either inferiorly or superiorly to obtain a portion of tubing free of perforation sites if leaking were to cause either deterioration of the venous image and/or if venous distention becomes more difficult.

#### Internal Jugular Vein (IJV) model

**Figure 2:**
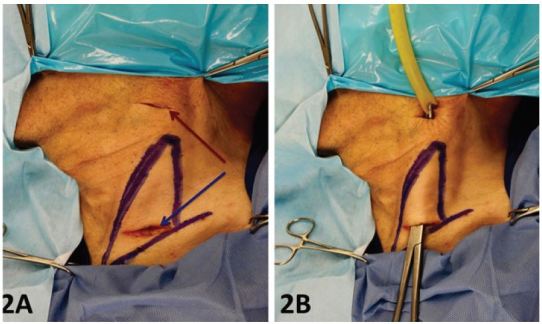
2A) Internal jugular vein model: The two heads of the sternocleidomastoid (SCM) are drawn (in purple) to demonstrate the anterior triangle of the neck. The first incision is made between the two heads of the SCM just above the supraclavicular line (blue arrow). The second incision is made at the submandibular line (red arrow) 2B) A long clamp is used to tunnel through the anterior triangle at the level of the fascia from inferior to superior incision points. A length of latex tubing is clamped and pulled through the tunnel to replicate the IJV. *IJV*, internal jugular vein

For the internal jugular vein (IJV) cannulation a similar technique is used. Make an incision, just above the clavicle between the two heads of the sternocleidomastoid muscle (SCM) and as close to the upper border of the clavicle as possible. (Figure 2A). The inferior incision is carried down to the level of the fascia just superficial to the IJV, which is visible in the incision site as a light blue structure. Care must be taken due to the fact that the IJV lies superficial in the space between the two heads of the SCM muscle. The superior incision is made just inferior to the mandible and in a straight line connecting the inferior and superior incision. A long clamp is inserted into the inferior incision and tunneled just superficial to the exiting the superior incision. The latex tubing is grasped at the upper incision and pulled so as to exit through the inferior incision (Figure 2B). As in the FV model, using a longer than necessary segment of latex allows the tubing to be pulled so that a fresh, non-penetrated segment can be used when necessary.

#### Fluid Infusion for Both Models

**Figure 3:**
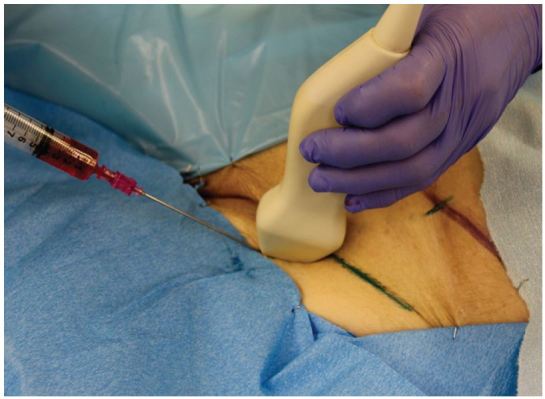
A drape is used to hide the exposed latex tubing and Toomey syringe. The model is now ready for implementation.

**Figure 4:**
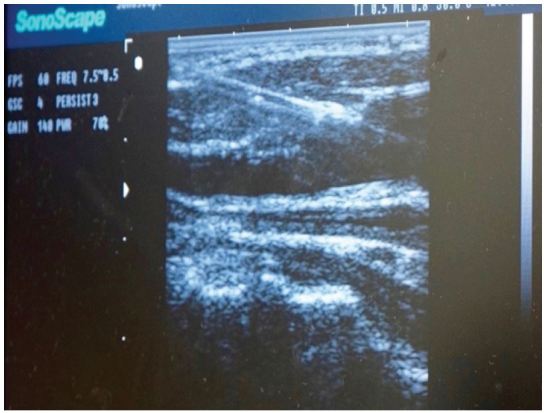
The latex tubing provides an anechoic, compressible vessel clone that is durable enough to withstand multiple “sticks” without extravasation.

A Toomey syringe filled with the fluid solution is fastened to the exposed open end of the tubing. In order to maximize the quality of US imaging, one should attempt to aspirate all air from the tubing prior to injecting fluid into the latex tubing. After aspirating, inject a solution of water mixed with 1ml red dye (approximately 20cc of fluid). After fluid infusion, both exposed ends of tubing and the Toomey syringe are hidden under the drape so as not to provide students with needle guiding cues (Figure 3). The simulated vessels are easily imaged and provide realistic anechoic, fluid-filled structures that can be compressed and cannulated (Figure 4). Students may then attempt US-guided identification and venous cannulation. An instructor, well versed in the procedure, is present to actively instruct and ensure proper technique. Once the vein is cannulated and fluid aspirated, guide wire insertion may be performed. If extravasation into the tissue space occurs after multiple cannulation attempts, the latex tubing can be pulled from the upper or lower prior incision providing a non-punctured region of latex tubing for cannulation.

### Educational Curriculum and Assessment

During the third-year surgery clerkship at our institution, all students participate in a fresh cadaver lab designed to teach the ABCs of trauma. This is a technical skill lab where students actively participate in chest tube insertion, endotracheal intubation, and CVA placement. After learning general principles in groups of 15, students were divided into groups of three to practice USG-CVA cannulation of the internal jugular and femoral veins. Upon completion of the educational session, students’ procedure knowledge and technical skills were individually assessed (post-test). Five weeks later, students were asked to return to the cadaver lab and their knowledge and technical skills were assessed again (retention-test). Student procedural knowledge was assessed with an 18-item checklist (Appendix A) that included components regarding indications, contraindications, and complications related to the procedure. Student technical skill was assessed using an 18-item checklist (Appendix B), which was developed by two of the authors experienced with USG-CVA placement.

#### Data Analysis

Wilcoxon ranked-sum testing for non-parametric samples was used to assess differences in population means. All analyses were performed using SAS statistical software (Version 9.2, Cary, NC, USA).

## RESULTS

A total of 56 students participated in this study. All 56 (100%) of the students completed a survey before and after the educational session. Mean post- and retention-test scores for technical skills performing USG-CVA (out of 18 maximum points) and number of correctly identified indications, contraindications, and complications for central venous lines are shown in Table. There was a significant difference between mean scores for pre- and post-test central venous line placement (2.7 vs 12.4, respectively, p<0.0001). There was also significant difference in the mean number of correct indications, contraindications, and complications of central venous line placement (1.5 vs 3.2, 0.8 vs 1.7, and 1.6 vs 2.7, respectively, all p<0.0001).

## DISCUSSION

During the past decade, numerous authors have noted a marked decline in formal procedural skills training during medical school.^7^,^12^–^14^ This is especially concerning for a procedure-rich residency such as emergency medicine, anesthesia, and general surgery, where numerous procedures such as CVC are performed and can lead to complications.^15^,^16^ The use of US has been shown to significantly decrease CVC complication risk, which may be further mitigated by improving procedural familiarity and competence through simulation education.^17^,^18^ In fact, the Agency for Healthcare Research and Quality has identified US-guided central venous placement as one of the practices in which strong evidence supports its widespread implementation.^19^ To ensure proficiency of this commonly performed procedure, US simulation models have been introduced to train venous access skills.^19^ Simulation-based medical education has proven to be a viable tool in CVC training and assessment.^18^ The fresh cadaver model improves upon the sim-man model by supplementing both tactile realism and maintaining the varied anatomic relationships found in live patients.^7^,^8^,^12^

Unfortunately, the fresh cadaver model introduces its own unique challenges. In the early years of using the fresh cadaver as a venous cannulation model, we found that after infusing approximately one liter of intra-venous fluid our cadavers became “bloated” as fluid extravasates out of the small vessels and lymphatics and into the interstitial space making the body less useful as the session proceeded. The result is distortion of normal anatomy and eventual deterioration of US imaging. One other author also describes this finding as a difficulty in the utilization of the fresh cadaver as a cannulation model.^10^ Similarly, in our own attempts to infuse fluid directly into cadaver vessels, we noted local fluid extravasation and subsequent deterioration of US imaging after only a few cannulation attempts. Our model improves upon this limitation, preserves cadaver imaging, and facilitates continued use of the cadaver for other procedures such as intubation, chest tube insertion, pericardiocentesis, etc.

The thin-walled latex tubing used our novel fresh cadaver model minimizes fluid extravasation and maintains venous distention despite numerous cannulation attempts. This tubing is a very close replica to the native vein, providing both a compressible structure and an US image accurately resembling the natural vein seen in the living patient. In our experience, depending upon the gauge of needle used, one can obtain up to 20–30 “sticks” with a 22-gauge needle and 5–10 “sticks” with an 18-gauge needle, which is used if guide wire insertion is required of the trainees. Tunneling a long segment of the tubing permits pulling the tube at one end providing a new segment for continued cannulation. (Figure 1B) In addition, the tube diameter can be varied so as to accommodate different cadaver body habitus. The objective is to have the tubing in its normal anatomic position without being able to palpate the tubing. This allows students to use landmarks without unrealistic visual or tactile clues to guide needle placement. Finally, tunneling the latex tubing directly on top of the vein gives the added value of retaining the anatomic relationships seen in the living patient. Incorporating latex tubing of variable diameters can enable trainees to experience difficult as well as simpler cannulations, thus grading the challenge according to experience.

## LIMITATIONS

Although this model appears to create a lifelike training simulation for CVC cannulation, the transference and retention of skills has been tested in a small group of students from one institution. In addition, our institution has an active well-supported Willed Body Program that provides donor cadavers at a very reasonable cost. Not every medical center will have this program available to them, and as a result cadaver and material cost are variable. In our study, we did not compare our novel model for CVC simulation to the standard fresh cadaver model. Another limitation of our model was not having a simulated carotid artery. This certainly would have added to the realism of our model and will be considered in future design modifications. Lastly, our cadaver model relies on instructors for successful implementation.

## CONCLUSION

The use of this novel cadaver model prevented extravasation of fluid, maintained ultrasound-imaging quality, and proved to be an effective educational model allowing third-year medical students to improve and maintain their technical skills.

## Supplementary Information





## Figures and Tables

**Table:** Mean (range) of pre- and post-test scores (N=56).

Item	Pre-test	Post-test	P-value
Central line placement*	2.7 (0–13)	12.4 (8–18)	<0.0001
Number of indications	1.5 (1–3)	3.2 (2–4)	<0.0001
Number of contraindications	0.8 (0–2)	1.7 (0–3)	<0.0001
Number of complications	1.6 (1–3)	2.7 (1–4)	<0.0001

* Out of 18 maximum points.
