# Supplementary material for: Introducing a Fresh Cadaver Model for Ultrasound-guided Central Venous Access Training in Undergraduate Medical Education
Source: West J Emerg Med. 2016 May 5;17(3):362–6. doi: 10.5811/westjem.2016.3.30069 (PMC4899071; doi:10.5811/westjem.2016.3.30069)
Supplement: Supplementary file 1 [file wjem-17-362-s001.pdf]

**Appendix A: Procedural Knowledge Checklist**

## Central Line Placement (Internal Jugular)

Indications:
1. Monitor CVP
2. Difficult peripheral vascular access in the multi-injured trauma patient
3. Delivery of concentrated electrolytes, i.e. potassium
4. Total Parenteral Nutrition
5. Plasmapheresis or long term antibiotics
6. Delivery of agents irritating to peripheral veins, i.e. amiodarone, phenytoin, mannitol
7. During cardiopulmonary resuscitation

Contraindications:
1. Local site infection or burn
2. Anatomic abnormalities
3. Coagulopathy

Complications:
1. Injury to artery
2. Arrhythmia
3. Air embolism
4. Thoracic duct injury (with left internal jugular)
5. Pneumothorax or hemothorax
6. Infection of cannulation site
7. Bleeding/hematoma
8. Venous thrombosis, pulmonary emboli

Total Points _____/18
