# Supplementary material for: Introducing a Fresh Cadaver Model for Ultrasound-guided Central Venous Access Training in Undergraduate Medical Education
Source: West J Emerg Med. 2016 May 5;17(3):362–6. doi: 10.5811/westjem.2016.3.30069 (PMC4899071; doi:10.5811/westjem.2016.3.30069)
Supplement: Supplementary file 2 [file wjem-17-362-s002.pdf]

Appendix B: Internal Jugular Vein (IJV) Central Line Cannulation Checklist

1. Prepare and drape the area:	**0**	**1**
2. Identify the correct transducer:	**0**	**1**
3. Identify the necessary equipment needed for the procedure:		
a. Some of the equipment necessary for procedure identified.	**0**	**1**
b. Most of the equipment necessary for procedure identified.	**0**	**1**
c. All of the equipment necessary for procedure identified.	**0**	**1**
4. Places US probe in the correct anatomic location for IJV localization	**0**	**2**
5. Identifies vein by its compressibility characteristics.	**0**	**2**
6. Places needle in correct angle to skin with bevel up	**0**	**2**
7. Aspirates as the needle advances towards the vein.	**0**	**2**
8. Obtains a flash back of fluid.	**0**	**3**
9. Removes needle from syringe in preparation for passing guide wire through the needle.	**0**	**2**
